# Fertility and Iron Bioaccumulation in *Drosophila melanogaster* Fed with Magnetite Nanoparticles Using a Validated Method

**DOI:** 10.3390/molecules26092808

**Published:** 2021-05-10

**Authors:** Fernanda Pilaquinga, Sofía Cárdenas, Doris Vela, Eliza Jara, Jeroni Morey, José Luis Gutiérrez-Coronado, Alexis Debut, María de las Nieves Piña

**Affiliations:** 1 Laboratorio de Nanotecnología, Escuela de Ciencias Químicas, Pontificia Universidad Católica del Ecuador, Avenida 12 de Octubre 1076 y Roca, Quito 17-01-2184, Ecuador; aneleaifos@hotmail.com (S.C.); enjara@puce.edu.ec (E.J.); 2 Department of Chemistry, University of the Balearic Islands, Cra. de Valldemossa Km. 7.5, 07122 Palma de Mallorca, Spain; Jeroni.morey@uib.es (J.M.); Neus.pinya@uib.es (M.d.l.N.P.); 3 Laboratorio de Genética Evolutiva, Escuela de Ciencias Biológicas, Pontificia Universidad Católica del Ecuador, Avenida 12 de Octubre 1076 y Roca, Quito 17-01-2184, Ecuador; dvela508@puce.edu.ec; 4 Department of Applied Physica Chemistry, Universidad Autónoma de Madrid, 28049 Madrid, Spain; josluguc@gmail.com; 5 CENCINAT, Universidad de las Fuerzas Armadas ESPE, Sangolquí 170501, Ecuador; apdebut@espe.edu.ec

**Keywords:** magnetite nanoparticles, chitosan, *Drosophila melanogaster*, iron bioaccumulation, fertility

## Abstract

Research on nanomaterial exposure-related health risks is still quite limited; this includes standardizing methods for measuring metals in living organisms. Thus, this study validated an atomic absorption spectrophotometry method to determine fertility and bioaccumulated iron content in *Drosophila melanogaster* flies after feeding them magnetite nanoparticles (Fe_3_O_4_NPs) dosed in a culture medium (100, 250, 500, and 1000 mg kg^−1^). Some NPs were also coated with chitosan to compare iron assimilation. Considering both accuracy and precision, results showed the method was optimal for concentrations greater than 20 mg L^−1^. Recovery values were considered optimum within the 95–105% range. Regarding fertility, offspring for each coated and non-coated NPs concentration decreased in relation to the control group. Flies exposed to 100 mg L^−1^ of coated NPs presented the lowest fertility level and highest bioaccumulation factor. Despite an association between iron bioaccumulation and NPs concentration, the 500 mg L^−1^ dose of coated and non-coated NPs showed similar iron concentrations to those of the control group. Thus, *Drosophila* flies’ fertility decreased after NPs exposure, while iron bioaccumulation was related to NPs concentration and coating. We determined this method can overcome sample limitations and biological matrix-associated heterogeneity, thus allowing for bioaccumulated iron detection regardless of exposure to coated or non-coated magnetite NPs, meaning this protocol could be applicable with any type of iron NPs.

## 1. Introduction

Nanotechnology has made it possible for biomedical applications and diagnostics to create, characterize, and modify the functional properties of nanoparticles (NPs) [1]. Inorganic NPs mediate between the molecular and solid states and combine physical and bulk phase properties with chemical accessibility in solution [2]. Thus, they are ideal elements for building nanostructured materials and devices with physical and chemical properties that are customizable. In addition, NPs have physical and chemical properties that differ from both the atom and the equivalent of the bulk counterparts. Magnetic NPs have a large surface area and exhibit quantum size effects, both of which drastically change some of the magnetic properties and exhibit superparamagnetic phenomena and magnetization quantum tunneling, as each particle can be treated as a single magnetic domain [3].

Iron NPs are especially useful in nanotechnology. This metal is an essential element that performs very important functions in living beings [4]; therefore, iron poisoning is rare [5]. Iron poisoning severity is related to the amount of ingested elemental iron [6]; since the human body does not have efficient mechanisms for excreting large amounts of iron, intoxication from a high intake (>60 mg kg^−1^) of this metal can be lethal [7]. Iron oxides are used as nanosized magnetic particles (i.e., maghemite [γ-Fe_2_O_3_] or magnetite [Fe_3_O_4_] with single-domain diameters of approximately 5–20 nm); magnetite (Fe_3_O_4_NPs) is a better choice in terms of biocompatibility [8]. Fe_3_O_4_ is a typical magnetic iron oxide with an inverse spinel structure [9], and at room temperature, its electrons can hop in the octahedral sites between 2+ and 3+ ion oxidation states, making magnetite an important element of the semi-metallic material class [10]. These magnetic NPs can be dispersed into suitable solvents with the proper surface coating, forming homogeneous suspensions called ferrofluids [11]. Ferrofluids constitute colloids in which magnetic NPs are distributed and stabilized in a liquid carrier. Such a suspension can connect with an external magnetic field and be positioned to a particular region, allowing for magnetic resonance imaging for medical diagnosis and AC magnetic field-assisted cancer therapy [12]. Current potential applications of magnetic NPs are continuously advancing and include guided drug and gene delivery [13], tissue engineering [14], magnetic resonance imaging [15], enzyme immobilization [16], and protein and metal adsorption [17].

NPs are sometimes coated with various materials to enhance their advantages, for example with chitosan (Ch). Among the most abundant polysaccharides, after cellulose and hemicellulose, is chitosan, which is biodegradable and non-toxic and is produced through deacetylation of chitin [18]. Free amino and hydroxyl groups are present in the chitosan that enable NPs to bind to different chemical groups and ions, facilitating different applications, such as protein and metal adsorption, directed delivery of drugs and genes, magnetic resonance imaging, tissue engineering, and immobilization of enzymes. In addition, this form of NPs could be used for the destruction of malignant cells in hyperthermia treatment [19]. However, few papers have focused on chitosan [20] and its nanotoxicology with magnetite NPs in in vivo tests [21,22].

Given the limitations of in vivo experimentation, the use of insects as a model organism is considered a valid starting point to extrapolate the findings to other living beings. Among insects, *Drosophila melanogaster* has a biological system suitable for the study and detection of chemical species, which has been determined after extensive study of its genetics [23]. *Drosophila* as a test system has several advantages: it is a eukaryotic organism with sexual dimorphism [24] (i.e., the male can be easily distinguished from the female), a large number of individuals can be obtained in a small space at low cost [25], and its life cycle lasts 10 to 12 days [26] at 25 °C and 60% relative humidity [27]. Thus, *Drosophila melanogaster* can be used to rapidly test for nanotoxicity and then ascertain its underlying mechanisms.

Some toxicity studies related to *Drosophila melanogaster* survival after Fe_3_O_4_NPs exposure have been carried out [28], but they cover iron bioaccumulation in fly eggs, not adult flies [29]. There are also reports regarding the effects of magnetite on fly longevity [30] and fertility [28,30,31]. Vecchio et al. [32] indicated the high toxicity of Fe_3_O_4_NPs for fertility in *Drosophila melanogaster*, but only at low concentrations (1.91–38.5 µg g^−1^). According to the toxicity ranking of metal NPs for *Drosophila*, non-coated Fe_3_O_4_NPs take third place, after silver and cobalt NPs, respectively [32], but bioaccumulated concentrations of each metal have not been studied. In other investigations [33,34] with eggs and male adult flies exposed to food containing AgNPs, NPs exposure was found to shorten their lifespan but not to lower their exposure to Ag ions (AgNO_3_). On the other hand, AgNPs of 20–30 nm displayed less toxicity than larger of 500–1200 nm, implying that there is a nanotoxicity scale for silver particles [35,36]. The lifespan of *Drosophila melanogaster* can therefore be used to examine NPs toxicity metrics, given the finding that its lifespan can be shortened by the total amount of ingested NPs but not by their total surface area.

Reactive oxygen species (ROS) have been identified as the primary cause of toxicity induced by nanomaterials such as TiNPs [37] and AuNPs [38,39]. The intracellular ROS level in *Drosophila* was found to have increased in this study after oral ingestion of different sizes of NPs. However, various NPs sizes had no impact on the development of ROS, suggesting that the total surface area of NPs is not a significant parameter for oxidative stress induction. Melanization and cuticle development defects were a consequence for adult *Drosophila melanogaster* fed with NPs, which indicates that *Drosophila* can also be used to research metabolic disorders caused by nanomaterials; nonetheless, further studies are needed to better understand pigmentation defects [38].

This study aimed to validate an analytical method for measuring iron bioaccumulation in *Drosophila melanogaster* flies fed with magnetite NPs regarding limits of detection and quantitation. Chitosan was used as a coating to compare iron concentration determination and verify its effect on fly fertility. Four different Fe_3_O_4_NPs concentrations (100, 250, 500, and 1000 mg L^−1^) were tested to determine changes in the number of offspring after exposure, as well as iron bioaccumulation.

## 2. Results

### 2.1. Fe_3_O_4_NPs and Ch- Fe_3_O_4_NPs Synthesis and Characterization

**Figure 1:**
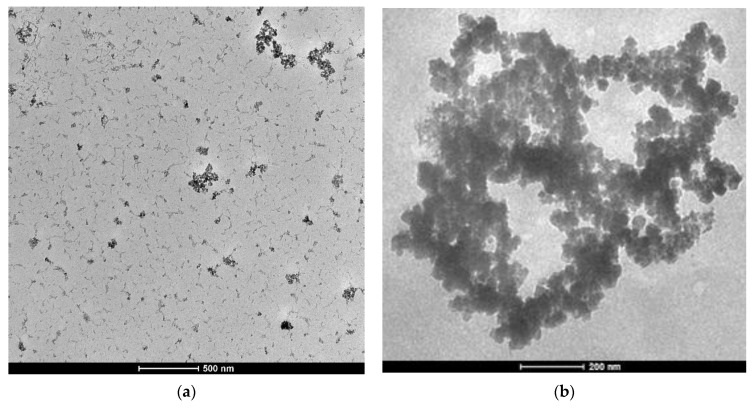

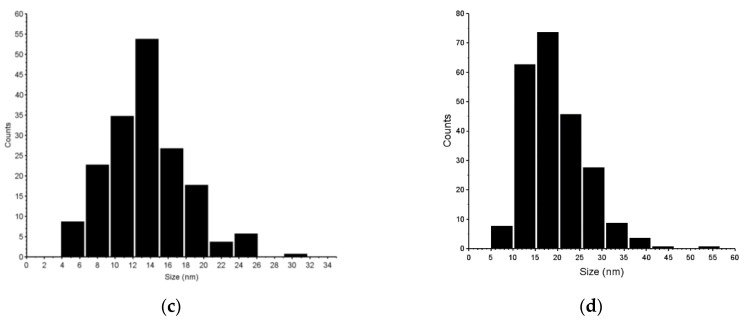
Magnetite NPs characterization: TEM image of Fe_3_O_4_NPs (**a**) Ch- Fe_3_O_4_NPs (**b**) and size distribution histogram Fe_3_O_4_NPs (**c**) Ch- Fe_3_O_4_NPs (**d**).

**Figure 2:**
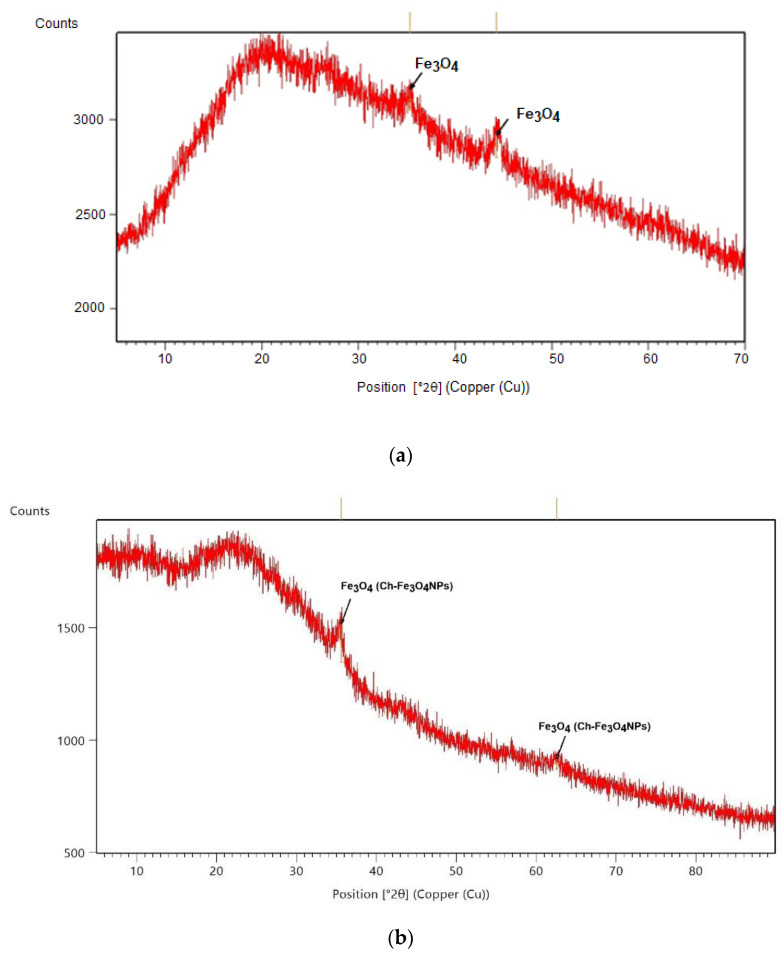
X-ray diffractograms: Fe_3_O_4_NPs (**a**) and Ch- Fe_3_O_4_NPs (**b**).

In Figure 1, the TEM image and size distribution of synthesized Fe_3_O_4_NPs and Ch-Fe_3_O_4_NPs can be observed. As can be seen in the TEM Figure 1a,b, due to the ferromagnetic nature of the magnetite structure, the NPs were not perfectly dispersed but tended to form some aggregates, which, in this case, had at maximum a diameter of approximately 600 nm. Obtained NPs were quasi-spherical, and their estimated average size were 13.96 ± 4.49 (Figure 1c) and 18.7 ± 5.20 nm (Figure 1d), respectively, as measured using the Fiji software. Magnetite crystalline nature was confirmed from the XRD analysis. A magnetite structure of Fe_3_O_4_NPs is also shown in the diffractogram in Figure 2; the bottom of the diffractogram curves correspond to the amorphous part of the glass that served as support, and of the amorphous phase of the extract. There are two diffraction peaks at 35.3° and 44.6° corresponding to magnetite formation on the (111) and (131) planes in Fe_3_O_4_NPs (Figure 2a) and in Figure 2b.

It is found that Bragg Reflection peaks at 36.06° coincide with the cubic phase of Fe_3_O_4_ (ICSD: 96012). The lattice parameter and highest intensity plane (113) are well matched and agree with other reported patterns. Hematite or metal hydroxides were not identified, which confirms the complete formation of Fe_3_O_4_.

**Figure 3:**
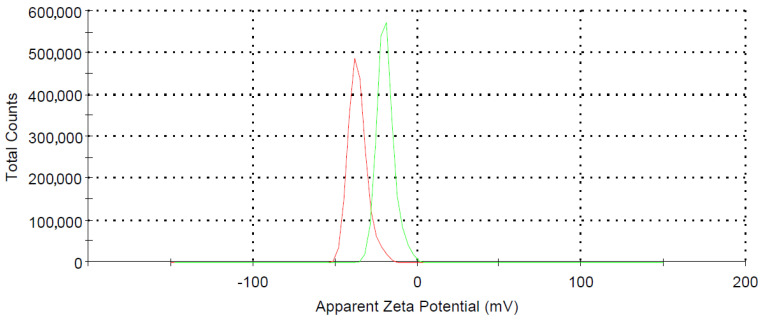
Zeta potential distribution: Fe_3_O_4_NPs (red) and Ch-Fe_3_O_4_NPs (green).

In Figure 3, the zeta potential distribution for Fe_3_O_4_NPs (red) and Ch-Fe_3_O_4_NPs (green) are shown. The value of −36.3 mV for Fe_3_O_4_NPs is compatible with the non-functionalization with a high-density negative charge [40]. The presence of chitosan on the surface of Ch-Fe_3_O_4_NPs modifies this value until −19.3 mV. The values of polydispersity index (PDI) were 0.28 and 0.34 respectively, demonstrating that the particle size distribution is uniform in both cases, as well as good suspension stability [41].

### 2.2. Fe_3_O_4_NPs’ and Ch-Fe_3_O_4_NPs’ Effects on Drosophila melanogaster Fertility

**Figure 4:**
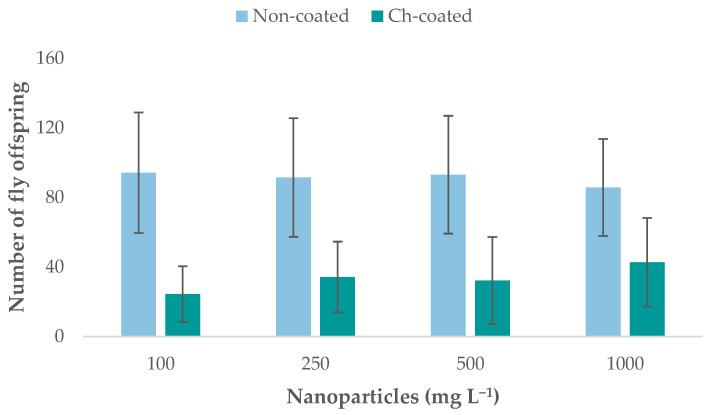
*Drosophila*’s offspring after exposure to Fe_3_O_4_NPs and Ch-Fe_3_O_4_NPs. Each column shows the average number of adult flies that emerged in each tested concentration, along with the standard deviation (*n* = 10).

The offspring of crossed flies exposed to Fe_3_O_4_NPs and chitosan-coated (Ch- Fe_3_O_4_NPs) were counted to estimate the effect of four NPs concentrations on the fertility of flies after exposure (Figure 4). The offspring of the control crosses (flies not exposed to NPs) were also counted.

The offspring of control crosses totaled 1197 flies. No statistical differences between the control crosses and crosses exposed to Fe_3_O_4_NPs were found; however, significant differences were observed between control crosses and crosses exposed to Ch-Fe_3_O_4_NPs. An analysis of variance (ANOVA) and Tukey’s HSD test showed significant differences (*p* > 0.05) between fertility of flies exposed to Fe_3_O_4_NPs and those exposed to Ch-Fe_3_O_4_NPs (Supplementary Materials, Tables SI-1.1 and SI-1.2). For non-coated NPs, offspring numbers corresponded to 942, 914, 930, and 857 flies for 100, 250, 500, and 1000 mg L^−1^, respectively. In the case of Ch-Fe_3_O_4_NPs, there were 244, 342, 322, and 427 offspring for 100, 250, 500, and 1000 mg L^−1^, respectively. Fertility of flies exposed to Ch-Fe_3_O_4_NPs, at all concentrations, was significantly lower than flies exposed to non-coated Fe_3_O_4_NPs.

### 2.3. Method Validation 

Overall, the validation of the method to quantitate iron in *Drosophila melanogaster* was satisfactory, as it showed that: (a) limits of quantification (LOQs) were 6.44 mg kg^−1^ and 16.9 mg kg^−1^ for the low and high concentrations, respectively (Table 1); (b) considering both accuracy and precision, the method was optimal for concentrations greater than 20 mg kg^−1^ (Table 2); (c) despite medium heterogeneity, iron dosage was proven to be in accordance with the desired exposure concentrations for this experiment (Figure 2). Please see Supplementary Materials, SI-2 for linear fit and confidence limits.

Blank measurements are displayed in Table 1; all of them were within the confidence limits. Minimal values that can be quantified with this analytical method are 6.44 mg kg^−1^ (low range) and 16.9 mg kg^−1^ (high range). These values were obtained by considering the volume and average mass used in the sample preparation procedure.

As seen in Table 2, fortifications with the matrix showed a high recovery after correcting all values with the average iron concentration present in flies (49.9 mg kg^−1^). In previous unpublished experiments, it was observed that in the absence of the matrix, iron adhered to crucible walls, causing lower recovery values and lack of reproducibility; by using a characterized matrix, this last issue was solved as demonstrated by the relative standard deviation, which was below 5% in all cases. Recovery values were considered optimum within the 95–105% range. At lower concentrations, recovery greater than 105% could be explained by the impossibility of obtaining homogeneous samples and the possibility that the flies used for this particular spiking had a higher biological iron content prior to exposure. Thus, it can be established that this method is valid at concentrations higher than 20.0 mg kg^−1^.

**Figure 5:**
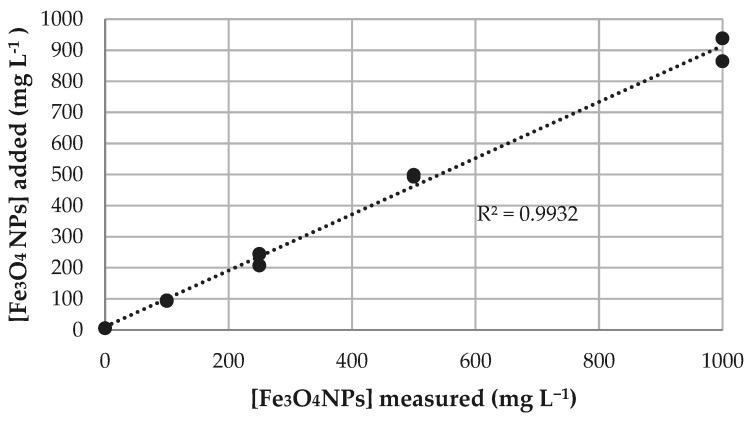
Correlation between added Fe_3_O_4_NPs and observed iron concentrations in the culture medium.

Prior to fly sample analysis, the iron recovery from the culture medium was assessed. First, each individual component of the culture medium was tested to determine its iron concentration (see Supplementary Materials, SI-3); as none was an important source of metal, we established a correlation between the added amount of NPs and the iron signal measured in the resulting mixture, shown in Figure 5. Small discrepancies were attributable to medium heterogeneity and the fact that added NPs were in a ferrofluid suspension. The obtained linear relationship demonstrates an adequate culture medium preparation and assures that flies were given the proper NPs dosage.

### 2.4. Iron Bioaccumulation in Drosophila Flies

**Figure 6:**
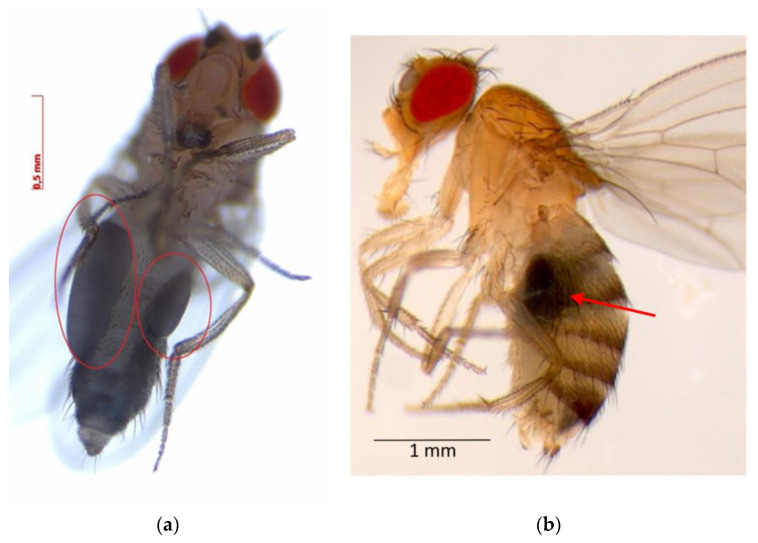
Iron bioaccumulation: Fe_3_O_4_NPs in the abdominal region of flies as observed with a stereomicroscope (**a**) ventral view and (**b**) lateral view.

After NPs exposure, emerged flies showed iron bioaccumulation in the abdominal cavity when observed with a stereomicroscope (Figure 6), as there were darkened regions around the midgut area. Iron concentration in these flies is shown in Table 3; all data were corrected with the blank’s concentration (49.9 mg Fe per kg of non-exposed flies).

**Figure 7:**
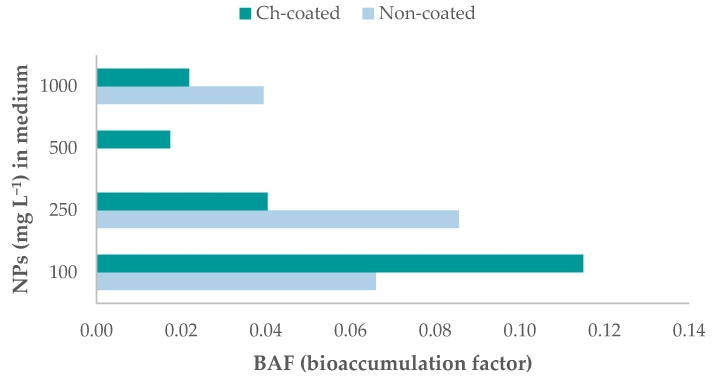
Bioaccumulation factor per nanoparticle concentration in culture medium fed to flies. Results shown for non-coated and chitosan-coated magnetite nanoparticles.

Having measured the iron concentrations in both exposed and non-exposed *Drosophila* and the matrix of exposure, we calculated the bioaccumulation factor (BAF). As seen in Figure 7, a trend emerged for Ch-Fe_3_O_4_NPs: doses with lower NPs concentrations corresponded to a higher relative buildup of the foreign material compared to doses with higher NPs concentrations in the culture medium. However, this trend did not appear in non-coated NPs assays.

## 3. Discussion

### 3.1. Fe_3_O_4_NPs’ and Ch-Fe_3_O_4_NPs’ Effect on Drosophila melanogaster Fertility

Our findings show that Fe_3_O_4_NP and Ch-Fe_3_O_4_NP exposure decreased *Drosophila melanogaster* fertility for all tested concentrations. The ANOVA showed that flies exposed to Ch-Fe_3_O_4_NPs had a significant decrease in fertility compared to flies exposed to Fe_3_O_4_NPs (*p* > 0.05). The range of fertility in flies exposed to Ch-Fe_3_O_4_NPs was 24.4–42.7 offspring while in flies exposed to Fe_3_O_4_NPs, it was 85.7–94.2; the highest fertility level was observed in non-exposed flies ($\bar{x}$ = 119.7). Significant differences (Supplementary Materials, SI-1.1) were observed when the fertility of Ch-Fe_3_O_4_NPs and Fe_3_O_4_NPs-exposed flies and the control group were compared, which demonstrated the negative effect of Ch-Fe_3_O_4_NPs on fertility. In the case of flies exposed to Fe_3_O_4_NPs, exposure to 1000 mg L^−1^ produced the lowest fertility level ($\bar{x}$ = 85.7), while the other treatments (including the control) showed higher fertility (Supplementary Materials, SI-1.1). In the case of Ch-Fe_3_O_4_NPs-exposed flies, there were significant differences among the control and both treatment groups (Supplementary Materials, SI-1.1); the lowest NPs concentration (100 mg L^−1^) produced the lowest fertility level ($\bar{x}$ = 24.4). These results are consistent with those reported by Asoufi et al. [30], who also exposed flies to Fe_3_O_4_NPs and identified an abnormal trend in the percentage of females in the fecundity stage. Variable behavior appeared in concentration ranges between 50 and 1000 mg L^−1^, with increased (50–100 mg L^−1^ and 200–400 mg L^−1^) and decreased reproduction rates (100–200 mg L^−1^ and 400–1000 mg L^−1^). This indicates that certain concentrations trigger a defense mechanism in *Drosophila melanogaster*, leading to the number of individuals being almost equal to that of the control population.

### 3.2. Iron Determination and Bioaccumulation

Petersen et al. point out the analytical challenges of bioaccumulation experiments in biological matrices [42]. Few studies have measured the accumulated residual iron in in vivo test specimens. For instance, Vega-Alvarez et al. [43] analyzed the effect of nanometric magnetite synthesized by coprecipitation and thermal decomposition methods on embryonic mortality of *Drosophila melanogaster*. Both types of preparation indicated an increase in mortality percentage: 0–42% (coprecipitation) and 0–46% (thermal decomposition); iron concentrations were estimated by extrapolating doses delivered during the microtransfer procedure. Affleck and Walker [44] offer a comprehensive description of techniques used to evaluate known and potential toxicants in *Drosophila*; however, they do not present information regarding the use of methods to assess metal bioaccumulation in a quantitative manner. One of the essential recommendations from Petersen et al. [42] is to make accurate quantitative measurements of nanomaterial concentration both in the biological species and in the matrix of exposure. Thus, it is imperative to evaluate the analytical method’s performance, especially regarding limits of detection (LOD), LOQ, and recovery. We propose a clear protocol to achieve this goal and obtained results with minimal bias. Our methodology included the determination of blank measurements to enable better differentiation of background iron concentration and NPs-attributable iron, as well as the demonstration of the correlation between added and measured metal concentration in the culture medium, to reduce artifacts from matrix heterogeneity that could compromise the interpretation of results.

Our findings show that iron bioaccumulation in flies is related to NPs concentration exposure, with one exception at 500 mg kg^−1^, where there was a notable decrease in Fe buildup. This concentration may be a threshold that prompts individuals to develop a defense mechanism. Henderson et al. [29] established that iron oxide NPs in concentrations of 10–100 µg mL^−1^ induce an innate immune response that leads to a survival effect of *Drosophila* larvae and adults. Our results are partially consistent with this hypothesis, as at 100 mg L^−1^ Ch-Fe_3_O_4_NPs, the number of adults decreased, and the highest BAF of all assays occurred. Mehta et al. [31] showed that iron-rich environments result in *Drosophila*’s midgut accumulation of iron-binding protein (ferritin), thus enabling the organism to confine high quantities of this element at this location. This is consistent with the accumulation sites observed in the current study.

Previous research has reported that toxicity observed by low fertility is also related to NPs size, which is the main feature that allows NPs to easily permeate the cell membrane and induce a biological response [45]. Gorth et al. [35] found that smaller NPs (20–30 nm) have a less toxic effect compared to those of 100 nm and 500–1200 nm because structurally, they do not accumulate within vital organs and thus do not cause a reduction in internal dose administration; however, they also pointed out a higher accumulation of smaller NPs at non-vital sites. Chen et al. [28] used doses of 300 and 600 µg g^−1^ of Fe_3_O_4_NPs sized 15 nm. Their results showed a 10% decrease in adult pupation and emergence rates when the highest dose (600 µg g^−1^) was administered; they concluded that oral exposure doses threaten all stages of *Drosophila melanogaster*’s development from oogenesis to emergence in adults. In this context and considering that our study used NPs of 15.8 ± 7.3 nm in diameter, we can deduce that their small size allowed for slight iron bioaccumulation in *Drosophila* individuals, but it did not interfere with vital processes, evidenced by the offspring number not being significantly different in the control group.

Fe_3_O_4_NPs and Ch-Fe_3_O_4_NPs are not lethal to *Drosophila melanogaster* as is the case with other metals. For instance, Gorth et al. [35] exposed *Drosophila* to silver NPs at different concentrations and sizes. The results showed that concentrations of 100 mg L^−1^ and a size range of 20–30 nm are lethal for adult eggs and flies; for this reason, the bioaccumulation test was performed only with a concentration of 10 mg L^−1^, which is 10–100 times lower than the concentrations of Fe_3_O_4_NPs tested in our experiment. Wu et al. [46] worked with calcium phosphate NPs and showed similar results to ours despite working with very low concentrations (3 ppm); they found that certain phosphate phases induce a boost in *Drosophila melanogaster* viability, although it is not clear why. This suggests that there may be other factors to consider during experimental design beyond NP size and concentration. Lankveld et al. [47] reported lower accumulation in rat tissue with smaller AgNPs, which contrasts with the findings from Gorth et al. [35]; the difference may be attributable to the exposure route, as Lankveld et al. [47] injected NPs into the specimens, while Gorth et al. [35] administered them orally. Other studies have demonstrated that a coating modifies the NP’s surface charge and thus the interaction it may have with the organism. Feng et al. [48] demonstrated differences in uptake, toxicity, distribution, and clearance of iron oxide NPs administered by injection to rats that depended on both NPs size and coating, even at low doses (1.5 mg Fe/kg). Furthermore, Jiang et al. [49] found that a positively charged surface, such as one coated with chitosan, enhances electrostatic interaction with *Drosophila*’s midgut, which in turn facilitates cellular uptake. Considering that these results were obtained with particles of about 140 nm in diameter, and the current study used a smaller diameter, it is feasible that both factors (smaller size and chitosan coating) promote Ch-Fe_3_O_4_NPs’ absorption and elicit a biological response that results in a decrease of fertility at all tested NPs concentrations.

## 4. Materials and Methods

### 4.1. Fe_3_O_4_NPs and Ch-Fe_3_O_4_NPs Synthesis and Characterization

Fe_3_O_4_NPs were synthesized using a coprecipitation method [50]. A 50 mL solution containing 0.30 g of FeCl_3_·6H_2_O (97%, Loba Chemie Mumbai, India) and 0.14 g of FeCl_2_·4H_2_O (98%, BDH Chemicals, London, UK) was prepared. This solution was heated at 60 °C ± 1 °C for 10 min; afterward, 50 mL of 2.5M NaOH (98%, Fisher Scientific, Waltham, MA, USA) were added dropwise. Helium gas was used to provide an inert atmosphere. A precipitate was obtained and washed with 10 mL of deionized water and then with 10 mL of acetone twice. Finally, the Fe_3_O_4_NPs were washed three times for 10 min with 10 mL of absolute ethanol (Scharlau 99%, Senmanat, Spain) in a Branson 3510 ultrasonic bath (Branson, Brookfield, CT, USA) at a frequency of 40 kHz. Prior to use, Fe_3_O_4_NPs suspension media was changed from ethanol to water by performing three washes with deionized water. Ch- Fe_3_O_4_ NPs initial reaction mixture contained: 50 mL of FeCl_3_·6H_2_O 0.32 M with 50 mL of FeCl_2_·4H_2_O 0.2 M and 50 mL chitosan 0.25%. This mixture was heated at 50 °C for 10 min with constant stirring before adding 20 mL of NH_4_OH 20% *v*/*v* (Merck, Darmstadt, Germany) dropwise. Helium was also used to provide an inert atmosphere for 20 min. Obtained NPs were separated with the help of a Nd-Fe-B magnet (Supermagnete, Gottmadingen, Germany). Subsequent washing and activation steps were performed as described above.

All synthesized NPs were characterized by means of transmission electron microscopy (TEM, FEI Tecnai G2 Spirit Twin, Hillsboro, OR, USA) and X-ray diffraction (XRD) (PANalytical Empyrean, Almelo, The Netherlands). For TEM, a FEI Spirit Twin with LaB6 filament was used, operating at 80 kV. The diffractometer had a θ-2θ configuration (Bragg-Brentano geometry) and was equipped with a Cu X-ray tube (Kα radiation λ = 1.54056 Å) operating at 40 kV and 40 mV; the analyzed sample was previously dried on a microscope slide at 40 °C to avoid any thermal degradation. A compensation for the 0.5 mm formed layer was provided in the alignment of the diffractometer sample holder device. The particle size distribution was determined by dynamic light scattering using a Malvern Zetasizer ZS90 model (Malvern Panalytical Ltd., Malvern, UK) at 25 °C and a concentration of 1 mg mL^−1^. The polydispersity index (PDI) was obtained simultaneously with the particle size.

### 4.2. Fe_3_O_4_NPs’ and Ch-Fe_3_O_4_NPs’ Effect on Drosophila melanogaster Fertility

The fertility of *Drosophila melanogaster* flies (Oregon strain) was assessed after exposure to four concentrations (100, 250, 500, and 1000 mg L^−1^). The flies were orally exposed by adding the corresponding concentration of NPs to banana culture medium [51], which was then fed to the flies. Ten crosses (five females × five males) were done for each concentration of NPs as well as control crosses without NPs. The parents were removed on the sixth day of the experiment to avoid overlapping of generations. The offspring were counted for 14 days after the first emerged fly. The parents and crosses were maintained in controlled conditions: 22 °C (±1 °C), 48% humidity, 12-h photoperiod, and banana culture medium.

### 4.3. Iron Determination and Method Validation

Iron quantification was performed as per the AOAC 999.11 protocol using the AAnalyst 400 atomic absorption spectrophotometer (Perkin Elmer, Waltham, MA, USA). Between 0.3 and 0.4 g of samples were weighed and calcined at 450 °C in a furnace (Comecta, Barcelona, Spain) for eight hours. Later, ashes were treated with 0.1 N HNO_3_ (69%, Loba Chemie) for one hour, filtered, and diluted to the mark in a 25 mL volumetric flask. Samples consisted of each component of the culture medium, culture medium as a whole, and flies exposed and unexposed to Fe_3_O_4_NPs and Ch-Fe_3_O_4_NPs. 

To assure the quality of results, a partial validation was performed. Uncertainty calculation was ruled out because of certified reference material unavailability. For the analysis, 1000 mg Fe L^−1^ of certified standard solution (Labequim, Puebla, Mexico) were used to prepare the calibration solutions and spiked samples. Two curves were constructed for low (0.125, 0.500, 0.750, 1.000, and 1.500 mg L^−1^) and high (1.000, 2.000, 3.000, 4.000, and 5.000 mg L^−1^) ranges to enable more flexibility regarding sample concentrations; 50 mL of each dilution were prepared and maintained at 4 °C until needed. All calibrations were a function of atomic absorption spectrometry (AAS) measurements versus concentration, after which a linear regression was performed to estimate upper and lower confidence limits (UCL and LCL, respectively) (Equation (1)). The value of T was selected from Student’s t-distribution at n-1 degrees of freedom (2.569) and a 95% confidence level:

(1) $$y=\;\left(a\pm T\times \varepsilon_{a}\right)\;+\;\left(b\pm T\times \varepsilon_{b}\right)\;\times \;x$$

The LOD refers to the lowest quantity of a substance that can be distinguished from the absence of that substance (a blank sample) within a stated confidence limit (generally 1%), while the LOQ is the smallest quantity that can be assured to be present. Thus, the LOD and LOQ were determined from the signals produced from six blank samples (0 ppm of iron) per calibration curve (12 in total). All these solutions were prepared according to AOAC 999.11 protocol. LOD and LOQ values were obtained by multiplying the standard deviation of all blanks by 3 and 10, respectively.

To assess accuracy and precision, four spiked samples were prepared with the following amounts of added analyte: 18.5, 20.0, 45.0, and 60.0 mg kg^−1^. Between 0.2 and 0.3 g of *Drosophila melanogaster* flies were used as the matrix. Afterward, 1 mL of each iron fortification was added and evaporated without boiling prior to calcination at 450 °C for eight hours. Ashes were dissolved with 10 mL of nitric acid 0.1 N and transferred to a 50 mL volumetric flask. Duplicate samples, except iron fortifications at 1.85 mL, were analyzed by AAS. Accuracy was determined as percent of recovery (Equation (2)), while precision was assessed by means of relative standard deviation (%RSD). For these parameters, acceptance criteria were established as 100 ± 5% for recovery, and RSD < 10%.

(2) $$Recovery\left(\%\right)\;=\frac{Instrument\;reading}{True\;value}\times 1000$$

Considering that the assays for accuracy and precision required the use of *Drosophila melanogaster* flies as the matrix, it was necessary to assess their existing iron content. This was done with eight independent samples, all of which underwent the same aforementioned procedure, without the iron solution addition.

### 4.4. Iron Bioaccumulation in Drosophila Flies 

Quantification of iron accumulated in the abdominal cavity of exposed flies was estimated in the offspring obtained in the assay described in Section 2.4 as per the AOAC 999.11 protocol. Prior to analysis, flies were isolated for 24 h after emerging, immobilized in a CO_2_ chamber, and stored at 0 °C. Average iron concentrations in flies and the medium were later used to estimate the BAF as the quotient of metal concentration in banana culture medium and in *Drosophila* bodies [42,52].

### 4.5. Statistical Analysis

To compare the fertility of flies exposed to the Fe_3_O_4_NPs and Ch-Fe_3_O_4_NPs treatments and the control, a one-way ANOVA, Duncan’s test, and Tukey’s HSD test were performed. The analysis was carried out using the SPSS statistical package, version 26.0. Significant difference was accepted when *p* < 0.05. 

## 5. Conclusions

This study validated an analytical method for the determination of the bioaccumulation of iron in *Drosophila melanogaster* flies fed with magnetite NPs; this protocol proved to be versatile for the direct measurement of metal content in flies, as it can be applied with confidence for both coated and non-coated NPs to obtain a valid correlation between the toxicant and the observable effect. Our findings highlight that iron bioaccumulation is associated with NPs concentration. All tested concentrations of chitosan-coated NPs produced a toxic effect, which decreased the fertility of exposed *Drosophila* flies; however, for non-coated NPs, only the 1000 mg L^−1^ concentration produced such an effect. In addition, it was determined that a dose of 500 mg L^−1^ produced no effect for both coated and non-coated NPs, resulting in values close to that of the control population for both fertility and iron bioaccumulation, suggesting this concentration can be used with any type of coating for in vivo studies with this species.

## Figures and Tables

**Table 1:** Blank measurements used for the estimation of limits of detection (LOD) and quantification (LOQ).

		Low Range	High Range
1		−0.007	−0.062
2		−0.021	−0.034
3		−0.023	−0.065
4		−0.028	−0.091
5		−0.032	−0.085
6		−0.015	−0.098
LOD	mg L^−1^	0.027	0.071
	mg kg^−1^	1.93	5.07
LOQ	mg L^−1^	0.090	0.237
	mg kg^−1^	6.44	16.9

**Table 2:** Accuracy and precision for iron quantitation in *Drosophila melanogaster* samples.

[Fe]_added_ (mg kg^−1^)	[Fe]_read_ (mg kg^−1^)	Recovery (%)	$\bar{X}$	%RSD
20	32.9	164.7	161.18	3.14
	31.5	157.6		
45	40.4	89.8	92.75	4.50
	43.1	95.7		
60	57.7	96.2	95.20	1.56
	56.5	94.1		

**Table 3:** Bioaccumulated iron in Drosophila melanogaster exposed to non-coated and chitosan-coated magnetite nanoparticles. All concentrations were corrected with blank measurements.

NPs (mg L^−1^) in Medium	[Fe in Flies] (mg kg ^−1^)	
	Exposure to Fe_3_O_4_NPs	Exposure to Ch-Fe_3_O_4_NPs
100	6.6 ± 3.0	11.5 ± 3.9
250	21.4 ± 1.0	10.1 ± 1.7
500	<Limit of quantification	8.70 ± 1.7
1000	39.5 ± 2.7	21.9 ± 5.6

## Data Availability

The data presented in this study are available on request from the corresponding author.

## Supplementary Materials

The following are available online. Table SI-1.1: Statistical analysis of fly fertility after exposure to Fe_3_O_4_NPs, Ch-Fe_3_O_4_NPs, and control; Table SI-1.2: Tukey’s HSD test results for control, Fe_3_O_4_NPs, and Ch-Fe_3_O_4_ NPs groups; Table SI-2.1: Linear fit, upper, and lower confidence limits (UCL and LCL) for each iron concentration within the studied ranges; Table SI-3.1: Iron concentrations in individual culture medium components.

## Sample Availability

Samples of the compounds are not available from the authors.
