# Supplementary material for: Fertility and Iron Bioaccumulation in Drosophila melanogaster Fed with Magnetite Nanoparticles Using a Validated Method
Source: Molecules. 2021 May 10;26(9):2808. doi: 10.3390/molecules26092808 (PMC8126126; doi:10.3390/molecules26092808)
Supplement: Supplementary file 1 [file molecules-26-02808-s001.zip › molecules-1112349-supplementary-proof done.pdf]

Supplementary Materials: The following are available online: Table SI-1.1: Statistical analysis of fly fertility after exposure to Fe_3O_4NPs, Ch-Fe_3O_4NPs, and control; Table SI-1.2: Tukey's HSD test results for control, Fe_3O_4NPs, and Ch-Fe_3O_4 NPs groups; Table SI-2.1: Linear fit, upper, and lower confidence limits (UCL and LCL) for each iron concentration within the studied ranges; Table SI-3.1: Iron concentrations in individual culture medium components.

SI-1: Fertility: Statistical analysis

Table SI-1.1 Statistical analysis of fly fertility after exposure to Fe_3O_4NPs, Ch-Fe_3O_4NPs, and control

NP Concentration	Fe_3O_4NPs						Ch-Fe_3O_4NPs					
	N	Mean	Std. Deviation	F	Sig.	Duncan's test [a]	N	Mean	Std. Deviation	F	Sig.	Duncan's test [a]
0 ppm	10	119.7	37.256	1.5	0.208	A	10	119.7	32.511	22.9	0,00	A
100 ppm	10	94.2	34.653			A	10	24.4	16.001			B
250 ppm	10	91.4	34.1			A	10	34.2	20.357			B
500 ppm	10	93	33.86			A	10	32.2	25.046			B
1000 ppm	10	85.7	27.92			B	10	42.7	25.456			B

[a] Means with the same letter are not significantly different ($p < 0.05$)

Table SI-1.2. Tukey's HSD test results for control, Fe_3O_4NPs, and Ch-Fe_3O_4NPs groups

Groups (I)	Groups (J)	Mean Difference (I-J)
Control group	Fe_3O_4NPs	28.625*
	Ch-Fe_3O_4NPs	86.325*
Fe_3O_4NPs	Control group	−28.625*
	Ch-Fe_3O_4NPs	57.700*
Ch-Fe_3O_4NPs	Control group	−86.325*
	Fe_3O_4NPs	−57.700*

* The mean difference is significant at the 0.05 level.

SI-2: Linearity assessment for iron quantification by atomic absorption spectrophotometry

Table SI-2.1 shows that the linear fit was acceptable for both concentration ranges. It also indicates that as iron concentration increases, confidence limits broaden; iron range between 3.000 and 5.000 mg L^{-1} showed the highest UCL.

Table SI-2.1. Linear fit, upper, and lower confidence limits (UCL and LCL) for each iron concentration within the studied ranges

Low Range $y = x - 1 \cdot 10^{-4}$ $R^2 = 0.9996$			High Range $y = x + 2 \cdot 10^{-4}$ $R^2 = 0.9982$		
[Fe]std mg L^{-1}	UCL	LCL	[Fe]std mg L^{-1}	UCL	LCL
0.000	0.030	-0.030	0.000	0.235	−0.234
0.125	0.159	0.090	1.000	1.424	0.576
0.250	0.289	0.211	2.000	2.614	1.387
0.500	0.549	0.451	3.000	3.803	2.197
1.000	1.068	0.932	4.000	4.993	3.007

1.500	1.587	1.413	5.000	6.182	3.818

SI-3: Determination of iron concentration in culture medium components

Components of the culture medium, which formed the basis of the Fe_3O_4NPs suspensions, are shown in Table SI-3.1. Most of the components had an iron content below the LOD, except the jelly; although the jelly proportion in the medium was very low, this was not an issue in the final culture medium.

Table SI-3.1. Iron concentrations in individual culture medium components

Ingredient	[Fe] (mg kg^{-1})
Banana	<LOD
Lemon	<LOD
Jelly	17.6 ± 0.34
Yeast	<LOD
Nipagin	<LOD
